# Patients with severe mental illness and their carers’ expectations for GPs’ communication skills: a qualitative approach in Spain

**DOI:** 10.3399/BJGPO.2023.0124

**Published:** 2024-03-20

**Authors:** Juan Andrés Ramos-Ruiz, Alejandro Pérez-Milena, Carmen Noguera-Cuenca, Antonina Rodríguez-Bayón, Beatriz Ruiz-Díaz

**Affiliations:** 1 Multiprofessional Teaching Unit of Family and Community Care Jaén North – Northeast Andalusian Healthcare Service, Andalusia, Spain; 2 El Valle Primary Care Center, Andalusian Health Service, Jaén, Spain; 3 Department of Psychology, University of Almeria, Almeria, Spain

**Keywords:** general practitioners, severe mental illness, caregivers, communication, qualitative research, primary healthcare

## Abstract

**Background:**

Effective communication with GPs (General Practitioners) enables higher rates of patient satisfaction and adherence to treatment plans. People with severe mental illness (SMI) and their caregivers present unique characteristics that present difficulties in the GP–carer–patient communication process.

**Aim:**

To explore the expectations of patients with SMI and their caregivers regarding GPs’ communication skills in primary care consultations.

**Design & setting:**

Face-to-face interviews, using focus group methodology, which were undertaken in southern Spain.

**Method:**

Forty-two participants took part in 21 paired semi-structured interviews with an average duration of 19±7.2 minutes. Information was audio-recorded and transcribed verbatim. Qualitative content analysis was undertaken, obtaining a codification in categories by means of triangulation.

**Results:**

Four themes emerged from the analysis. Theme 1 was interviewer communication characteristics. The ability of GPs to use a language that was colloquial and adapted to each person was perceived as a determinant of the quality of care provided. An empathetic attitude, low reactivity, and efficient time management were the most valued communication skills. Theme 2 was telemedicine: telephone consultation and video consultation. The telephone consultation was perceived as a useful tool to care for people with SMI. Video consultation was valued as a requirement in isolated rural areas. Theme 3 was the role of the caregiver during the clinical interview. The caregiver was considered by the patients as an ally who improves the clinical interview. Theme 4 was the perceived barriers and facilitators during the clinical interview. The continuity of care, defined by a low turnover of GPs, determined the quality perceived by those who required care.

**Conclusion:**

Themes emerging from this study have suggested that people with SMI require an inclusive, collaborative, and personalised approach in the care they receive from the public health system. Improved communication between GPs and patients with SMI is an essential requirement for quality medical care.

## How this fits in

The narratives of people who live with SMI are marked by painful experiences of disconnection with the social world that further increase the affliction and adversity of those who have the mental disorder. GPs trained in communication skills makes it possible to offer positive, differentiating care to these people, providing solutions based on collaborative care management between primary and secondary care.

## Introduction

People with severe mental illness (SMI) present a set of psychotic or pre-psychotic symptoms that make it difficult for them to understand reality and relate to others. This condition has negative repercussions in different areas of their personal lives (education, employment, social relationships) and can pose a risk to their lives.^1^ The health systems of countries that promote comprehensive care offer treatment adapted to the specific needs of these patients and their caregivers, respecting their individual needs and their interdependence.^2^ The underlying idea of this approach is that health services are provided based on the needs of the population, devoting more resources to the health care of those patients with the most serious and demanding conditions.^3,4^

The complexity in managing these conditions, including major depression, bipolar disorder, and schizophrenia, is a challenge for healthcare providers in both urban and rural areas.^5,6^

The current health reality, both in the public and private spheres, imposes certain limits that condition care for people with SMI such as efficient management, waiting times, number of people treated, biomedical response based on pharmacological treatment as well as absence of physical places for communication and sociability. It would be advisable to make these limits more flexible, in order to offer more immediate and personalised treatment that is closer to the expectations of each patient.^7^

In this context, it is necessary to use communication skills that allow GPs to create a climate of trust and provide effective support to patients.^8^ There are specific communication skills related to each phase of the clinical interview: initiation (quality and respectful reception); information gathering (open questions and verbal facilitation techniques); explanatory phase (concretisation and negotiation techniques); resolution phase (agreement on problems and their solutions); and closure (check understanding and make notes for future appointments). Both verbal and non-verbal communication are important in this doctor–patient relationship.^9^

Effective communication increases the satisfaction of professionals and users, adherence to treatment and clinical efficiency (fewer complementary tests and referrals to secondary care).^10^ It has also been shown to reduce the hyperutilisation of primary care consultations.^11^ We can therefore affirm that communication skills during the care interaction between doctor and patient are an essential tool.^12^ These skills acquire special relevance when the patient has an SMI.^13^

A new aspect to study is the skills necessary for telephone care for these patients, which is on the rise after the COVID-19 pandemic and which has contributed to overall inequity of psychiatric service provision.^14^ This remote attention could improve medical accessibility but hinders the relationship by suppressing non-verbal communication.

In a complementary way, the biopsychosocial approach that characterises family medicine facilitates a comprehensive therapeutic approach to the patient with SMI. This model of clinical care allows us to understand how suffering, illness, and disease are affected by multiple levels of organisation, from the social to the molecular. On a practical level, it is a way of understanding the subjective experience of the patient as an essential factor for accurate diagnosis, health outcomes, and humane care.^15^

There are previous studies that have explored those desirable primary care quality indicators to provide health care to people with SMI.^16^ In Spain, the experiences of relatives who live with people with SMI have been explored.^17^ Our research team has investigated the influence of the companion — an informal caregiver — in primary care consultations and communication skills of GPs.^18^

However, the expectations of patients and caregivers regarding communication processes with the medical professionals who care for them have not yet been sufficiently investigated. This study obtained information through qualitative techniques on the perspectives and needs of the patient with SMI and the person who cares for them during the clinical interview with GPs.

## Method

### Design and setting

Qualitative methodology with semi-structured interviews was used to explore the expectations of patients with SMI and their informal caregivers about the communication process during medical consultation. This methodology is suitable for this study owing to the exploratory nature of the research questions, which seek to reveal expectations and perceived needs on communication skills for GPs.

This article conforms to appropriate qualitative reporting guidelines.^19^

### Participants and recruitment

People with SMI and their caregivers participated. Twenty-one paired semi-structured interviews, using focus group discussion, were conducted to explore the communication expectations of people who request health care around the SMI in primary care. Participants were recruited from January 2021–June 2022. The study was carried out in the province of Jaén, in southern Spain. Purposive and convenience sampling was carried out by key informants (physicians, psychologists, or social workers who knew the patients and caregivers), in urban and rural areas.

### Data collection

**Figure 1.**
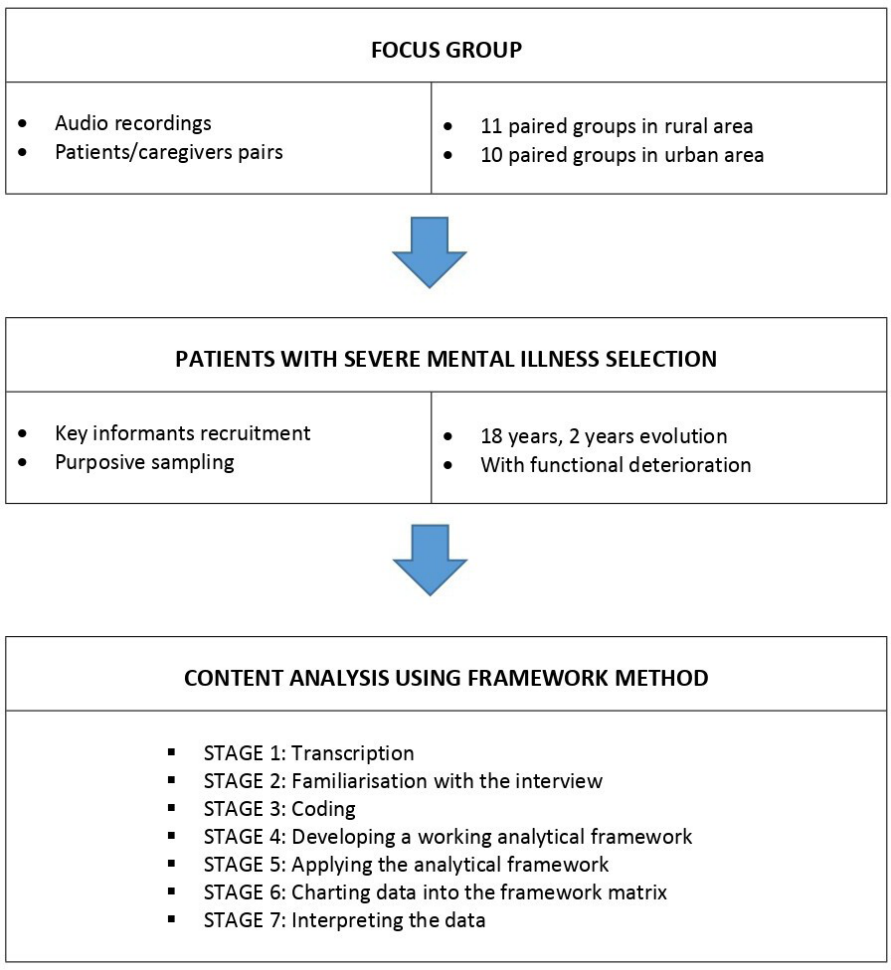
Flowchart of the qualitative study according to SRQR recommendations.

Face-to-face interviews by patient–caregiver pairs were carried out by JR and BR, with experience in semi-structured clinical interviews and following the agreed topic guide (Figure 1) designed by three members of the research team (AP, BR, and JR), according to the aim of the study. Interviewers had no prior professional contact with the people interviewed by them. These interviews began with a framing, introductory sentence, and an open-ended question, asking participants to express their experiences and expectations regarding the communication abilities of GPs who provide their health care.

### Qualitative analysis

All interviews were audio-recorded and transcribed verbatim with the prior consent of the patients and their caregivers. A content analysis of them was carried out, complying with the seven stages of framework approach described by Ritchie and Spencer, and Gale *et al*,^19,20^ and in accordance with the Standards for Reporting Qualitative Research (SRQR) recommended by O'Brien *et al* ^21^ (Supplementary Box S2).

AP and JR had previous experience in qualitative research studies.^22^ CN, owing to her role as a psychology professor at the University of Almeria, was aware of the need to explore expectations within this group. AR contributed her expertise as an established researcher in communication and health. BR had previous experience in conducting and transcribing clinical interviews. All the interviews were read in full by three members of the research team (AP, CN, and JR), who independently subjected them to preliminary coding using the triangulation technique. In subsequent meetings, the coding was refined and the final designation of the definitive topics was agreed on.

Patients who were decompensated or with some acute discomfort did not participate. To guarantee the ability of the people with SMI who participated in the study to understand and construct their own narrative, the World Health Organization Disability Assessment Schedule (WHODAS 2.0)^23^ was administered to all patients. The questionnaire measures the degree of health and disability, and allows the clinician to conceptualise the individual’s disability. In this study, disability is considered in people with SMI from a moderate to extreme degree (≥1.5 points).

Segmentation criteria were chosen to collect all possible opinions, using heterogeneity criteria to select different profiles of patients and caregivers. The heterogeneity criteria used were sex; diagnosis and disability, moderate to severe, assessed with the WHODAS 2.0 questionnaire; Apgar test^24^ to measure family function; and the Zarit Burden Interview to measure burden of the carers.

## Results

Twenty-one patient–caregiver paired interviews were carried out with a total of 42 participants and a mean duration of 19 minutes (± SD 7.2) (range 5–33 minutes). The patients had diagnoses of schizophrenia (38%), bipolar disorder (31%), and major depression (31%), with a mean age of 58.8 years (± SD 13.1). Sixty-three per cent were women, 32% were disabled, and 25% had family dysfunction. The caregivers were first-degree family members (50% spouses, 31% sons and/or daughters, 13% siblings, and 6% parents). Fifty-three per cent were men and the mean age was 52.9 years (± DS 16.9). Sixty per cent showed caregiving burden and 32% perceived family dysfunction. The characteristics according to an urban or a rural area are shown in Table 1. The data collected are presented in four categories and 10 subcategories (Table 2) corresponding to the topics of the interview script, the hypotheses generated, and the explanatory framework.

**Table 1.** Characteristics of study sites and participants

		Interview duration, minutes	Sex, female	Mean age, years	Family dysfunction^a^	Disability^b^	Caregiving burden^c^
**Rural zone**	Patient	21.8±2.5	57.1%	62.7±9.6	42.9%	28.6%	—
	Caregiver		66.7%	52.5±15.4		—	83.3%
**Urban zone**	Patient	16.8±9.7	66.7%	55.8±15.1	11.1%	33.3%	—
	Caregiver		33.3%	53.2±18.9	22.2%	—	44.4%

^a^Apgar test. ^b^World Health Organization Disability Assessment Schedule (WHODAS 2.0). ^c^Zarit Burden Interview.

**Table 2.** Categories and subcategories

Categories	Subcategories
1. Interviewer communication characteristics	1.1. Language understandable and adapted to patients and carers 1.2. Empathetic attitude and low reactivity 1.3. Time management
2. Telemedicine: telephone consultation and video consultation	2.1. Telephone consultation during the COVID-19 pandemic 2.2. Useful aspects of telephone consultation 2.3. Video consultation with psychiatrist
3. The role of the caregiver during the clinical interview	3.1. Facilitating attitude of the caregiver during the interview 3.2. The caregiver as a guarantee of therapeutic compliance
4. Perceived barriers and facilitators during the clinical interview	4.1. The high turnover of professionals as a barrier to communication 4.2. The figure of the nurse as a facilitator of communication

Following the framework approach,^20^ four main themes emerged from the analysis:

- Interviewer communication characteristics;
- Telemedicine: telephone consultation and video consultation;
- The role of the caregiver during the clinical interview;
- Perceived barriers and facilitators during the clinical interview.

### Interviewer communication characteristics

- **Language understandable and adapted to patients and carers**

Both patients and caregivers positively valued the application of a culture of care based on dialogue, where the effort by GPs who care for them to adapt the scientific language to more colloquial language is appreciated. The use of simple, clear, and friendly speech with the person with SMI became a basic need:

*'Sometimes, you miss that the person who gives you that information, that is, the family doctor, uses a language adapted to us. Because many times we leave the medical center saying: "What has this man told me?”.*' (56-year-old woman, caregiver of a patient with major depression, intense overload and family dysfunction, rural environment)

*'I tell him: explain it to me better, I don't understand. Because doctors have a special way of speaking and sometimes, we don't understand them and other times we do.*' (59-year-old woman with bipolar disorder and disability, urban environment)

*'When my GP asks me: "How’s the run-run going?", I know he’s referring to the intrusive and repetitive thoughts that sometimes pop up in my head. However, when foreign GPs come in, they don't understand this terminology.'* (49-year-old woman with major depression, rural environment)

- **Empathetic attitude and low reactivity**

Empathy and low reactivity are two essential characteristics of a good clinical interviewer. The empathetic professional is capable of not only putting themselves in the place of the person who is reporting a problem, but also of making that person feel heard and understood. On the other hand, the low reactivity of GPs in consultation allows listening to the experiential story of the patients, without premature interruptions, and paying full attention.

Participants with SMI reported perceiving a higher degree of empathy and low reactivity from those GPs they know. They commented that these qualities are less present in sporadic consultations such as those in emergency services:

*'My family doctor talks to me as if I didn't have a disability. I feel that he understands me and listens to me like he does with his other patients. Because, when my illness is under control, I am a normal person.'* (47-year-old woman with bipolar disorder, urban environment)

*'My GP gives me enough time to express myself; I don't feel like she’s in a hurry. However, I believe that in the Emergency Department, this doesn't happen because the doctors don't know me well enough.'* (51-year-old man with major depression, rural environment)

- **Time management**

Generosity of time spent by GPs takes on a leading role: without space or time, professionals cannot listen attentively and, therefore, therapeutic work is at risk. Caregivers positioned themselves as observers of the clinical interview between GP and patient, and often expressed the need for more time allotment for these consultations:

*'After waiting a long time, we go in for a consultation and they only spend five or six minutes … The doctor needs to be attentive, check the medication carefully, chat a little, and let you tell your doubts.'* (48-year-old man, caregiver of person with major depression, moderate overload, urban environment)

*'The waiting times in the emergency room are distressing, I get nervous, I have to go out into the street. You spend hours in the waiting room and the doctors don't call you. And then, when you enter, you don't have time to explain everything you need.'* (65-year-old man with bipolar disorder and disability, urban environment)

### Telemedicine: telephone consultation and video consultation

- **Telephone consultation during the COVID-19 pandemic**

During the months of the COVID-19 pandemic, in which face-to-face consultation was not possible, both patients and caregivers adapted to the telephone consultation on most occasions. The degree of satisfaction with them was high. Its problem-solving capacity and the reduction of waiting times stood out:

*'The last appointment, due to the COVID pandemic, was not in person. It was by phone … And she answered the questions that her family doctor asked her … There was no need for her to be face-to-face either … because she was calm and well.'* (62-year-old man, caregiver of person with bipolar disorder, poor family function, urban environment)

- **Useful aspects of telephone consultation**

The participants chose telephone assistance as the preferred consultation modality in specific situations such as mild or moderate relapses, treatment adjustments, and as a guarantee of access to check-ups in those patients from rural areas:

*'It would be very useful for decompensations or doubts, if we had prompt attention, on the same day, a telephone to speak directly with our family doctor, and many doubts could be resolved and we would avoid going to the emergency service.'* (47-year-old woman with bipolar disorder, urban environment)

However, for situations considered urgent or serious, participants viewed face-to-face interviews as a necessity:

*'If the reason for consultation was not serious, the telephone method was useful. But when we are really unbalanced, we need someone to look you in the eye, to know you, and to explain things to you face to face.'* (49-year-old woman with major depression, rural environment)

- **Video consultation with psychiatrist**

In rural areas that were far from the consultation with psychiatry (secondary health), the demand for video calls emerged as a useful communication tool for annual check-ups. With this method, patients and caregivers could share their narratives and come face to face with their psychiatrist. For this video consultation, they proposed the GP as a moderator. Agility, ease of access, and the guarantee of continuity of care were some of the aspects that made video consultation attractive:

*'We live in a rural mountain area, more than 100 kilometers from the Psychiatry service … Most of the annual check-ups with Psychiatry could be carried out by videoconference and our GP could be present to help resolve doubts. Also, just as the telephone consultation works, why can't the video consultation work?'* (54-year-old man, major depression, rural environment)

### The role of the caregiver during the clinical interview

In most settings, the caregiver stood as an ally during the clinician–patient interview. This offered bidirectional support that helped both GPs and the patient to improve the exchange of information and, therefore, mutual understanding.

- **Facilitating attitude of the caregiver during the interview**

A patient told how their sister and main caregiver helped them to express themselves and remembered the stories about the evolution of their condition when they were in front of their GP:

'*Sometimes I haven't slept well, I'm tired or I get nervous when my doctor is in front of me. If it weren't for my sister accompanying me, I wouldn't be able to remember the things I want to tell my GP and she helps me remember them.'* (56-year-old woman with borderline personality disorder and major depression, urban environment)

- **The caregiver as a guarantee of therapeutic compliance**

A caregiver described how they accompanied their brother to the consultation to listen to the explanation of their GP and, once at home, to remind them of the medication administration and dosage plan:

*'His doctor already knows me as well as he knows him and he thanks me for accompanying him to the consultation, because that way I can find out more about the changes that he indicates in the medication. There are times when my brother is compensated and he understands it easily, but there are other times when he needs me to be aware of what his doctor explains to him.'* (54-year-old woman, caregiver of a person with schizophrenia, rural environment)

### Perceived barriers and facilitators during the clinical interview

- **The high turnover of professionals as a barrier to communication**

Owing to the instability of medical staff in rural areas, patients felt that staff were unaware of their experiences and, therefore, did not feel understood. GPs who did not know patients often transmitted insecurity and mistrust during the clinical interview:

*'In the public system consultation, each time a different doctor sees you, and no one knows you. That is the reason to go to a private doctor. Because, in the private clinic, the same doctor always sees you and he already knows my sister. Sometimes, before telling him anything, he already knows what is happening to her.*' (57-year-old woman, caregiver of a person with schizophrenia, rural environment)

- **The figure of the nurse as a facilitator of communication**

Sometimes, when the high turnover of GPs meant that patients with SMI did not perceive that they were known, the figure of the nurse stood as a facilitator in the doctor–patient clinical interview:

*'I am lucky that my nurse has known me all my life. If I ever come in and my doctor is not there, she tells the substitute doctor my whole story. Because she has known me forever and knows how to understand what is happening to me.'* (55-year-old man, schizophrenia, disability, urban environment)

*'We need a GP who listen to us and know us to be able to tell him how we sometimes feel, feelings and very personal thoughts … When our doctor is not there, our nurse comforts us.'* (48 year-old woman, major depression, intense overload, rural environment)

## Discussion

### Summary

This study was conducted to find what patients with SMI and their carers felt during the communication process with GPs. The results of the research have shown that a majority of patients with SMI want collaborative health care^25^ between their family members, their social environment, and the healthcare system itself, where the GP is the main focus.

Patients with SMI and their caregivers demand a collaborative communication model with the clinicians who care for them. The results obtained have emphasised the role of the family doctor in promoting an adequate relationship with that population subgroup. Both patients and caregivers have high expectations in the quality of the communication they establish with their doctor. Communication training of health professionals is essential to promote a fluent transmission of information between patients, caregivers, and GPs.^26^

The use of colloquial language that is enriched with the expressions of each geographical area (localisms) provides greater therapeutic power to the discourse of the attending physician.^27^ Feeling listened to, being able to relate their experiences without fear of being prejudged and receiving cordial and empathetic treatment^28^ are the most valued communication characteristics of GPs. On the other hand, the main factor suggested for improvement is the high reactivity in the speech of those who care for them, which is usually related to less time spent during the clinical visit actively listening^29^ to the patient’s report.

Thus, the most valued communication skills of GPs by this group are as follows: mastery and use of a language adapted to the person with SMI; empathy and low reactivity during the clinical interview; and efficient time management. The study also found that video consultation appears as an expectation of care improvement for those people who live in rural and isolated environments. In addition, the caregiver is positioned as an ally in the clinical act, facilitating the flow of information between doctor and patient. Finally, the high turnover of professionals in primary care is identified as the main perceived barrier^30^ during the clinical encounter. The nursing professional was found to fulfil a facilitating function of communication.

In this study, patients proposed communication spaces that generate a safe context, away from impositions. They require that their honest opinion be taken into account and that their decision be respected, maintaining a horizontal transmission of information between sender and receiver. This reinforces some of the previous conclusions published by Pattyn *et al*.^31^ In the same way, they request that their narratives of affliction, suffering, overload, or hopelessness are recognised.^15^

In relation to the caregivers of people with SMI, we have detected that they are concerned in the first instance about the perceived helplessness in situations that require a more immediate approach (review before relapses, consultations on pharmacological treatment adjustments) and propose the use of telephone consulting as an effective and desired communication alternative.^32^

Video consultation appeared as a desirable option, especially in rural settings where check-ups with a specialist involve travelling many kilometres away; this is in line with previous research such as that of Casey *et a*l.^33^

Consistent with the findings of Pereira *et al*, the participants of the present study have proposed health policies that integrate effective efforts to maintain a consistent staff of professionals who care for them, especially in rural areas with difficult coverage.^34^

### Strengths and limitations

This study has explored, for the first time in Spain, the perspectives of patients with SMI and their caregivers regarding the communication barriers of the GPs who care for them. Their expectations for improvement are useful for generating future health programmes that help transform health systems.^30^ The results have illustrated the importance of the GP in the comprehensive care of patients with SMI and their caregivers. The skills to communicate closely and directly with these patients allow a greater degree of satisfaction for them.

Patients could potentially have modified their stories to avoid being especially critical of the healthcare system or their GPs. However, the fact that the semi-structured interview was carried out by a health professional with experience in it was a desirable factor. Taking these last two aspects into account, we decided that the interviews should be carried out by a healthcare professional with experience in qualitative methodology and close to the environment of the interviewed patients and caregivers.

In accordance with the idiosyncrasy of qualitative methodology, the findings of this study cannot be extrapolated to populations that differ from the reference population. However, this study makes it possible to lay the foundations for improving health care in environments with similar characteristics. We propose to replicate this research in other populations, in order to compare and improve the specific communication qualities required for the health care of people with SMI and their informal caregivers.

### Comparison with existing literature

To the authors' knowledge, this is the first Spanish study that is interested in the influence of GPs' communication skills on the quality of care perceived by people with SMI and their caregivers.

The desire to be informed correctly is a common feature in most patients. Giacco *et al* assessed the desire of people with SMI for information on their treatment and whether the desire for information is associated with therapeutic relationship and symptom severity.^35^

In another line of research, Aoki^36^ has been interested in the shared decision making (SDM) for adults with SMI. SDM is a communication process that may overcome traditional power imbalance and encourage changes among both users and professionals, and is defined by the following five phases: goal sharing; information sharing; deliberation; mutual agreement; and follow-up.

Previously, several authors have explored the relationship between SMI and physical health.^37,38^ For example, it is known that there is a higher mortality rate in people with SMI owing to cardiovascular diseases than in the general population.^39^ However, few previous investigations have been interested in learning about the communication skills of GPs who work with people with SMI.^13^

In a broader context, we can assert that there is a dissonance between clinical and patient conceptions of ideal care. In this study, the experiences and expectations of patients and their caregivers have been examined to understand the perception of quality of care. Nevertheless, further investigation is needed to identify how different physician–patient interaction styles influence the perceived quality of care and the time required to provide such care.^40^

### Implications for research and practice

Qualitative studies that reveal the expectations of the patients themselves and their caregivers regarding the characteristics of health care should be the starting point for designing, building, and improving the structure of future health systems. Many investigations have shown how approaches that neglect communication and quality social relationships only contribute to a worsening and stagnation of the situation of patients. Authors such as Seikkula,^41^ regarding the Finnish model of the Open Dialogue, place the question of communication as the central axis of health care.

The qualitative approach to the experiences of the people who have participated in this study and discovering their positions and knowledge, has allowed us to reflect and propose some action strategies around the issue of communication.^15^ Health systems should promote holistic care for the population, especially the population with SMI, taking into account the context, culture, meaning, and subjective internal experience, including the strengths and deepest fears of the person. This change could be implemented by utilising medical care models, such as the Co-Work-Care model,^42^ in order to improve communication and facilitate active dialogues between GPs, psychiatrists, caregivers, and the patients themselves.

## Data

The verbatim quotations with the expectations of the interviewees have been included in the Results section, preserving the privacy of the participants. It would be inappropriate to publish the personal data on which the conclusions of this research are based, as this would be outside the terms of the informed consent to participate.
